# Effect of Copper Doping on Electronic Structure and Optical Absorption of Cd_33_Se_33_ Quantum Dots

**DOI:** 10.3390/nano11102531

**Published:** 2021-09-28

**Authors:** Fengai Zhao, Shuanglin Hu, Canhui Xu, Haiyan Xiao, Xiaosong Zhou, Xiaotao Zu, Shuming Peng

**Affiliations:** 1 Institute of Nuclear Physics and Chemistry, China Academy of Engineering Physics, Mianyang 621900, China; fengaizh0506@163.com (F.Z.); xuch1209@caep.cn (C.X.); zlxs77@126.com (X.Z.); 2 School of Physics, University of Electronic Science and Technology of China, Chengdu 610054, China; hyxiao@uestc.edu.cn (H.X.); xtzu@uestc.edu.cn (X.Z.)

**Keywords:** DFT, CdSe quantum dots, oxidation state of Cu, doping, optical absorption

## Abstract

The photophysical properties of Cu-doped CdSe quantum dots (QDs) can be affected by the oxidation state of Cu impurity, but disagreement still exists on the Cu oxidation state (+1 or +2) in these QDs, which is debated and poorly understood for many years. In this work, by using density functional theory (DFT)-based calculations with the Heyd–Scuseria–Ernzerhof (HSE) screened hybrid functional, we clearly demonstrate that the incorporation of Cu dopants into the surface of the magic sized Cd_33_Se_33_ QD leads to non-magnetic Cu 3d orbitals distribution and Cu^+1^ oxidation state, while doping Cu atoms in the core region of QDs can lead to both Cu^+1^ and Cu^+2^ oxidation states, depending on the local environment of Cu atoms in the QDs. In addition, it is found that the optical absorption of the Cu-doped Cd_33_Se_33_ QD in the visible region is mainly affected by Cu concentration, while the absorption in the infrared regime is closely related to the oxidation state of Cu. The present results enable us to use the doping of Cu impurity in CdSe QDs to achieve special photophysical properties for their applications in high-efficiency photovoltaic devices. The methods used here to resolve the electronic and optical properties of Cu-doped CdSe QDs can be extended to other II-VI semiconductor QDs incorporating transition-metal ions with variable valence.

## 1. Introduction

Doping of semiconductor nanocrystals (NCs) or quantum dots (QDs) with transition-metal ions has attracted significant interest in the applications of lasers [1,2], biolabeling [3,4,5,6], light-emitting diodes [7,8,9], and optoelectronics devices [10,11,12,13,14]. In particular, the transition-metal ions that can introduce permanent, electrically, or optically active charges are desired dopants [2,15,16]. The active charge can be permanently introduced into the NC host lattice by incorporating a transition-metal ion having a variable valence [17,18]. By such doping, new energy levels could be introduced into the bandgap of the host NCs, which can exchange charges with the valence band or the conduction band, thereby significantly influence their electronic and optical properties. Copper exhibits variable valences (+1 and +2) and has become a promising doping element in II-VI semiconductor NCs to modify the electronic and optical properties for their desirable applications [2,17,19,20,21].

Several studies have been devoted to the incorporation of Cu impurity in II-VI semiconductor NCs (e.g., CdSe, CdS, and ZnSe) to introduce copper-related intragap emission property [18,22,23,24,25]. However, some questions still exist on the origin of dopant emission in these Cu-doped NCs. In materials such as CdSe NCs, a model system for studying the electronic and photophysical properties of doped II-VI semiconductor NCs [15,26,27,28,29,30], there is still disagreement on the oxidation state of Cu ion whether it presents a +1 or +2 valence state. Meulenberg et al. used soft X-ray absorption near-edge spectroscopy (XANES), X-ray photoelectron spectroscopy (XPS), and photoluminescence (PL) to examine the electronic and chemical structures of Cu ions dispersed in CdSe QDs and concluded that Cu ions had a +1 oxidation state [29]. The same +1 oxidation state for Cu in CdSe NCs was also assumed in Refs. [21,31]. However, Viswanatha et al. reported that Cu impurities in ZnSe/CdSe core–shell NCs exhibited a +2 oxidation state and served as a permanent source of optically active holes [17]. Brovelli et al. claimed that although the photoluminescence from their ZnSe/CdSe core–shell QDs was mainly attributed to Cu^2+^ ions, the possibility that some QDs might contain Cu^+^ ions could not be excluded [18]. To date, it is still unclear whether the oxidation state of Cu depends on its spatial distribution in CdSe QDs. This is mainly due to the difficulty of incorporating a well-controlled number of dopants at precise positions in small QDs. Therefore, it is important to understand the effects of the dopant environment on the photophysical properties of small-sized QDs. The second question is the location of Cu 3d orbitals within the forbidden band of II-VI NCs. Several research groups have reported that Cu 3d states were just above the valence band [24,32,33], while others claimed that Cu 3d orbitals were close to the conduction band [34]. The different positions of Cu 3d energy levels within the forbidden band will lead to very different recombination mechanisms of Cu impurity emission [32,35]. Thus far, it is unknown how the position of Cu 3d states varies with the dopant location in QDs either. Therefore, it is critical to investigate how the location of dopants in QDs influences the local atomic structure of QDs and thus modifies the state of Cu 3d orbitals as well as the optical properties of QDs.

To explore how the location of Cu dopants (at the surface or in the core region) affects the oxidation state of Cu ions and the position of Cu 3d orbitals in the energy levels of the Cu-doped semiconductor nanocrystals, we conduct a systematic density functional theory (DFT)-based study of the structural, energetic, electronic, and optical properties on the Cu-containing CdSe QDs. The Heyd–Scuseria–Ernzerhof (HSE) screened Coulomb hybrid functional was used in our DFT calculations, which is well known to predict the correct bandgaps of a wide range of materials successfully [36,37,38]. Wurtzite-based Cd_33_Se_33_ nanocluster with a core–cage structure was used as the host NC in this work, which has been experimentally demonstrated to be extremely stable and used as a model system for the II-VI semiconductor nanocrystals [30]. Previous extensive theoretical studies have been conducted focusing on its special atomic arrangement [39,40,41,42]. Therefore, the Cd_33_Se_33_ is a suitable host nanocrystal for the incorporation of copper impurity. Here, up to three Cu atoms are doped to substitute Cd atoms at different locations of a Cd_33_Se_33_ QD. Details of the dopant locations are described in the Methodologies Section. The corresponding structural distortions, energetics, electronic properties, and optical absorption spectra were investigated, respectively. The present work provides a theoretical perspective on how to control Cu dopants in CdSe NCs to achieve desired optoelectronic properties for their applications in high-efficiency photovoltaic devices.

## 2. Methodologies

All calculations were carried out based on the density functional theory (DFT) implemented in the Vienna ab initio simulation package (VASP) [43]. The ion–electron interactions were treated by the projector augmented-wave (PAW) approach [44,45]. The exchange-correction effects were described by the screened hybrid functional of HSE with a mixed approach combining Hartree–Fock (HF) and Perdew–Burke–Ernzerhof (PBE) [44]. Here, an HF:GGA mixing ratio of 0.33 and a range-separation parameter of 0.15 Å^−1^ were used, which successfully predicted the lattice parameters and bandgap for bulk wurtzite CdSe. The HSE setup was used for all the CdSe QDs in this work. A 1 × 1 × 1 Monkhorst-Pack mesh in the Brillouin zone was set for all CdSe QDs. Plane-wave cutoff energy of 350 eV was used, which resulted in good convergence of the total energy. To prevent spurious interactions between a QD and its periodic images, a vacuum spacing of at least 16 Å between the QD and its replicas was used in the simulation cells.

**Figure 1:**
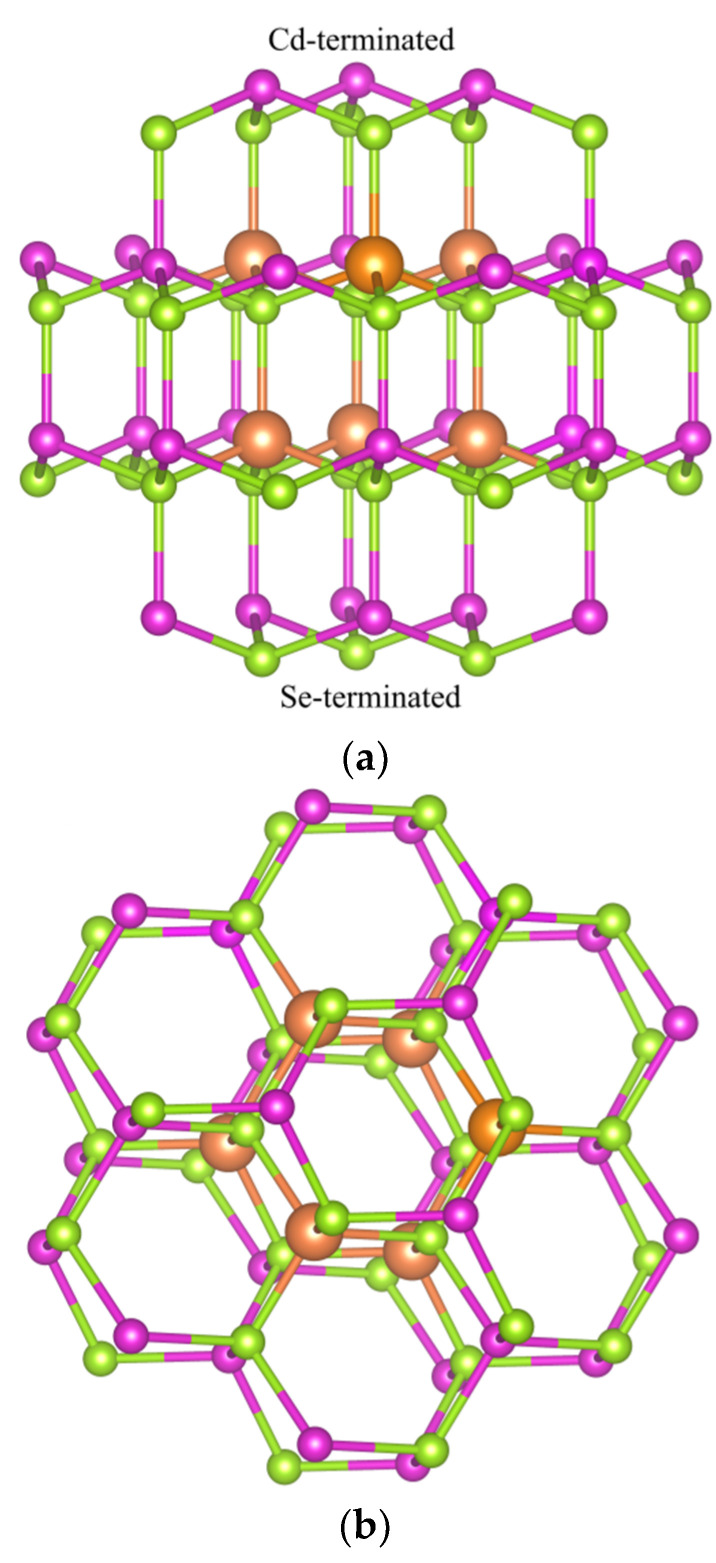
Geometrical structures of the unrelaxed Cd_33_Se_33_ quantum dots: (**a**) side view of the Cd_33_Se_33_ with the Se-terminated (bottom) and Cd-terminated (top) facets; (**b**) top view of Cd_33_Se_33_. The candidate doping sites (Cd sites) for a Cu impurity are labeled by small purple spheres for surface sites and large orange spheres for core sites, respectively; the Se atoms are represented by green spheres.

The Cd_33_Se_33_ was constructed on the basis of bulk wurtzite (WZ) lattice with a Cd–Se bond distance of 2.688 Å. Similar construction methods for modeling CdSe quantum dots have been widely used in previous publications [39,40,41,42]. In the unrelaxed Cd_33_Se_33_ (Figure 1), the wurtzite core was formed by stacking two Cd_3_Se_3_ rings (so the core is Cd_6_Se_6_), which were enclosed by the Cd-terminated and Se-terminated surface facets, respectively. The definition of Cd or Se termination followed that in Ref [41]. The non-core atoms were treated as surface atoms. It should be noted that the core and surface of a nanocrystal can be distinguished in experiments [39,46]. In the core region, a Cu dopant binds to four adjacent Se atoms before relaxation, which is the same as in a bulk CdSe. On the surface, a Cu dopant has two to four nearest Se atoms before relaxation. In the case of the core doping, Cu dopants can be close to the Cd or Se termination or both. Therefore, the incorporation of Cu atoms into the core region is divided into three cases: a Cu dopant substituting a Cd atom near the Cd-terminated facet is defined as type I substitution in the core region (marked as “C_I_”); a Cu dopant replacing a Cd atom near the Se-terminated facet is defined as the type II substitution in the core region (denoted as “C_II_”); a Cu dopant near the Cd-terminated and the other near the Se-terminated is defined as type III substitution in the core region (marked as “C_III_”). As for the surface substitution (S), all Cd sites on the surface were tested for Cu doping, and the lowest energy site was selected for further study.

## 3. Results and Discussion

### 3.1. Geometry Distortions of Cu-Doped Cd_33_Se_33_ Quantum Dots

**Figure 2:**
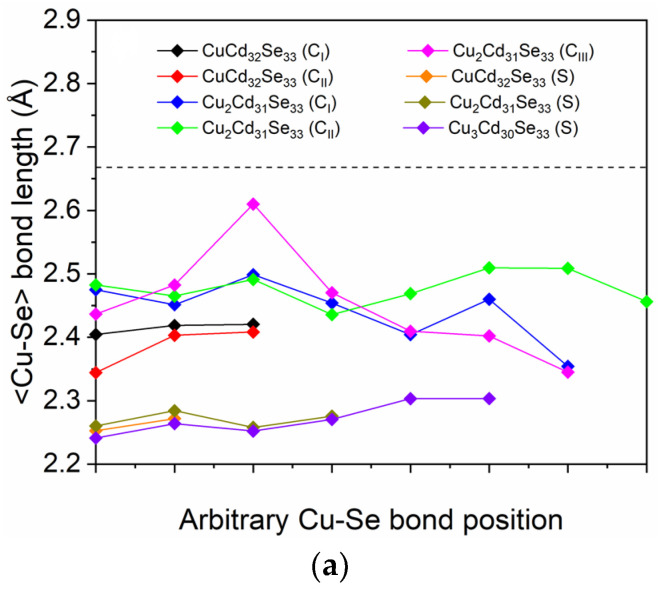

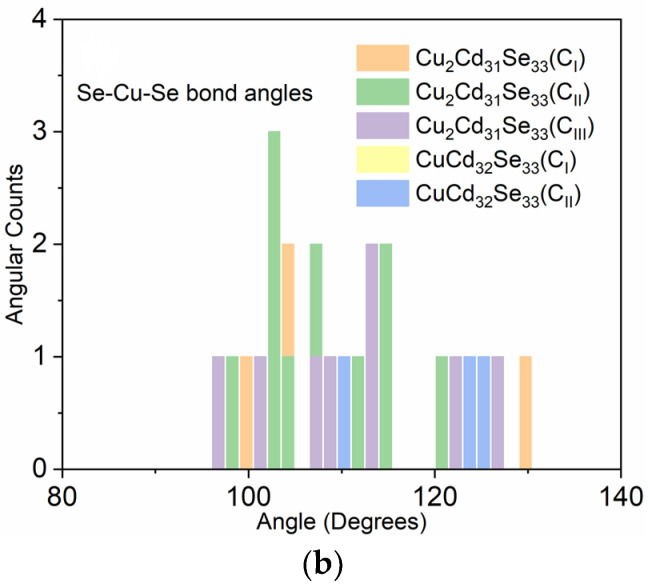
(**a**) The Cu-Se bond length in Cu-doped Cd_33_Se_33_ quantum dots, together with the Cd-Se bond length in pristine Cd_33_Se_33_ as indicated by the dashed line; (**b**) the bond-angle distributions of Se-Cu-Se when Cu atoms are doped in the core region of Cd_33_Se_33_.

After relaxation, the wurtzite core structure is retained in both pristine and Cu-doped Cd_33_Se_33_ QDs, although the surface structure has some degree of reconstruction. The average Se-Cd bond length in the relaxed pristine QD is determined to be 2.667 Å located between 2.678 Å in the core region and 2.657 Å in the surface region, which agree well with the value of 2.685 Å in other DFT calculations [39]. These values are slightly smaller than the Cd-Se bond length of 2.688 Å in bulk wurtzite CdSe, suggesting the lattice contraction effect in the nanocrystal. To further analyze the local environment for Cu atoms in Cd_33_Se_33_ QDs, the <Cu-Se> bond distance and <Se-Cu-Se> bond angles are summarized in Table 1 and graphically presented in Figure 2. For comparison, the <Cd-Se> bond distances and <Se-Cd-Se> bond angles in the pristine Cd_33_Se_33_ are also presented. It is found that the value of d<Cd-Se> in the pristine Cd_33_Se_33_ is larger than those of d<Cu-Se> in Cu-doped QDs, meaning that Cu doping results in shorter distances between a substituting site (Cd-site) and its neighboring Se atoms. This phenomenon may be attributed to the larger ionic radius of Cd than that of Cu [47]. The optimized Cu-Se bonds on the surface of the QD are about 6–8% shorter than those in the core, as indicated in Table 1 and Figure 2a. The 6–8% deviation is greater than the 0.8% difference between the core and surface region in Cd-Se bonds, indicating that the surface Cu induces a significant surface reconstruction. For the bond angle (∠), the ∠Se-Cu-Se between a Cu atom and its adjacent Se atoms in Cu-doped Cd_33_Se_33_ QDs are calculated in Table 1. The Se-Cd-Se bond angles in the core and surface regions for pristine Cd_33_Se_33_ (denoted as Cd_33_Se_33_ (C) and Cd_33_Se_33_ (S), respectively) are also presented, where Cd atom is the one replaced by Cu. The Se-Cu-Se bond angle in Cu-doped Cd_33_Se_3_3 (C) QDs ranging from 109.85° to 120.0° is generally larger than the original Se-Cd-Se bond angle of 109.04° in pristine Cd_33_Se_33_ QD, indicating the inward relaxation of Cu atoms in the core region. The phenomenon is similar to the case of Ag in CdSe QDs [48]. The <Se-Cd-Se> bond angle is calculated to be around 109.5° (sp^3^-like bond feature), and the coordination of substituted atoms (Cd atoms) in the core region of Cd_33_Se_33_ QDs is four. When a Cu atom incorporates into the core region, the coordination of substituted atoms became four-coordinated upon relaxation since <Se-Cu-Se> bond angles are around 120° (sp^2^ bond character). As for two Cu atoms incorporated in the core region, the distribution of <Se-Cu-Se> bond angles is similar to the case of <Se-Cd-Se> bond angles in Cd_33_Se_33_ (C), suggesting that the coordination of substituted atoms does not change after Cu atoms incorporation. However, in the surface-doped QDs, Cu dopants are finally bonded to two Se atoms after relaxation with the <Se-Cu-Se> bond angles ranging from 168.5° to 177.83° as shown in Table 1, which are different from <Se-Cu-Se> bond angles of 119.78° in surface-doped Cd_33_Se_33_ QDs. These differences in Cu-Se bonds (or <Se-Cu-Se> bond angles) of the surface and core region before and after Cu incorporation indicate that Cu doping in the QDs induces significant structural reconstruction.

### 3.2. Stability of Cu-Doped Cd_33_Se_33_ QDs

To investigate the stability of Cu dopants in Cd_33_Se_33_ QDs, the binding energy (E_b_) per Cu atom is calculated by using the following Equation [49]:

$$E_{b}=\left[\left(E_{\mathrm{undoped}}+{\;\mathrm{nE}}_{\mathrm{Cu}}\right)-\left(E_{\mathrm{doped}}+{\;\mathrm{nE}}_{\mathrm{Cd}}\right)\right]/n$$

Here, E_undoped_ and E_doped_ are the total energies of the Cd_33_Se_33_ and Cu-doped Cd_33_Se_33_ QDs, respectively; E_Cd_ and E_Cu_ are the total energies per atom in bulk Cd and Cu, respectively; n is the number of Cu dopants. The binding energy per Cu atom and total dopant binding energy are presented in Table 2. Similar to [22], here, a positive binding energy means that Cu is energetically favorable to replace Cd; meanwhile, a larger binding energy means that the Cu dopant has a stronger tendency to bind with the CdSe QDs. As shown in Table 2, the Cu binding energy decreases with the increasing number of Cu atoms for both surface- and core-doped Cd_33_Se_33_, meaning that the stability of the system decreases as Cu content increases. When three Cu atoms are introduced in Cd_33_Se_33_, the binding energy becomes negative. This result suggests that the number of doped Cu atoms in Cd_33_Se_33_ should be less than three per QD to maintain the system stability. In terms of Cu dopants at different locations of Cd_33_Se_33_, incorporation of one Cu atom at the surface (CuCd_32_Se_33_ (S)) generally results in a larger binding energy than that in the core region (CuCd_32_Se_33_ (C_I_) and CuCd_32_Se_33_ (C_II_)), suggesting that a Cu dopant prefers to segregate to the surface. For doping two Cu atoms, Cu_2_Cd_31_Se_33_ (C_III_) has a larger binding energy than the surface-doped Cu_2_Cd_31_Se_33_ (S), while Cu_2_Cd_31_Se_33_ (C_I_) and Cu_2_Cd_31_Se_33_ (C_II_) exhibit smaller binding energies, compared to Cu_2_Cd_31_Se_33_ (S). As discussed in the next section, these differences originate from the different oxidation states of Cu dopants in Cd_33_Se_33_ QDs. The results also demonstrate that the location and concentration of Cu atoms play important roles in system stability.

### 3.3. Oxidation State for Cu Dopants in Cd_33_Se_33_ Quantum Dots

**Figure 3:**
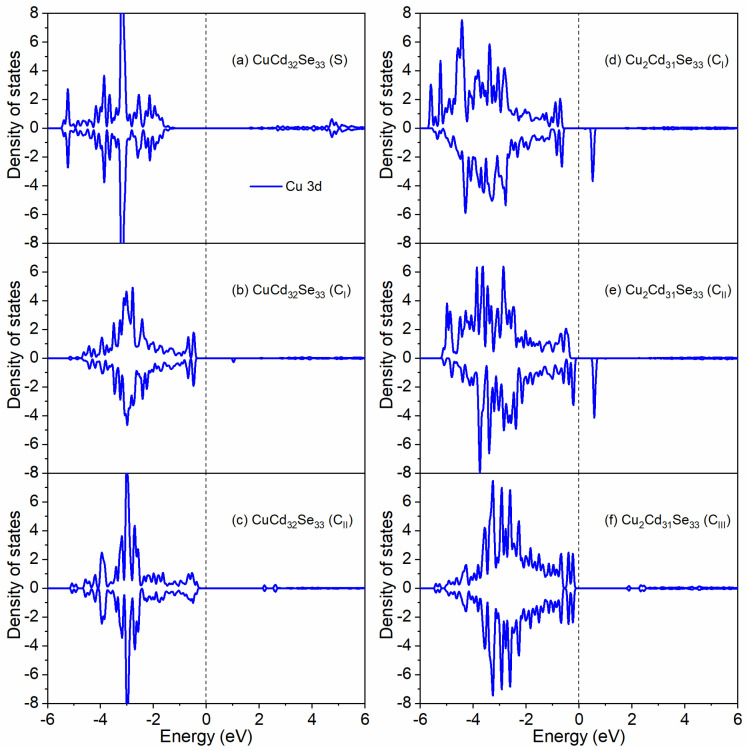
Orbital projected density of state (DOS) for Cu 3d in (**a**) CuCd_32_Se_33_ (S); (**b**) CuCd_32_Se_33_ (C_I_); (**c**) CuCd_32_Se_33_ (C_II_); (**d**) Cu_2_Cd_31_Se_33_(C_I_); (**e**) Cu_2_Cd_31_Se_33_(C_II_); (**f**) Cu_2_Cd_31_Se_33_(C_III_). Here, only the case of CuCd_32_Se_33_ (S) is presented since the spin-up and spin-down states of the Cu 3d orbitals are symmetric for all the surface-doped Cu:Cd_33_Se_33_. The Fermi level is located at 0 eV.

Generally, Cu^+^ has a fully filled 3d^10^ electron shell and exhibits non-magnetic properties, whereas Cu^2+^ has one unpaired electron in their 3d^9^ shell and thus behaves paramagnetically [17,18]. In our calculations, when Cu dopants are incorporated on the surface, the spin-up and spin-down states are symmetric for the Cu 3d orbitals (Figure 3a), indicative of a non-magnetic character. Therefore, Cu dopants in surface-doped Cd_33_Se_33_ present the +1 oxidation state. When one or two Cd sites in the QD core are replaced by Cu, the Cu 3d orbitals show distinct electronic features for different substitution locations, as presented in Figure 3b–f. In the case of doping a Cu atom in the core regardless near the Cd-terminated or Se-terminated surface (CuCd_32_Se_33_ (C_I_) or CuCd_32_Se_33_ (C_II_), as shown in Figure 3b–c), the density of states (DOS) of the Cu 3d states shows similar symmetric characters as those of surface-doped one (CuCd_32_Se_33_(S)), indicating that the +1 oxidation state of Cu is presented in these systems. The Cu 3d orbitals in Cu_2_Cd_31_Se_33_ (C_III_) (where one Cu dopant is close to the Cd termination and the other is near the Se termination) also show non-magnetic DOS distributions and thus the Cu^+^ electronic character, as shown in Figure 3f. However, when both Cu dopants are near the Cd termination (Cu_2_Cd_31_Se_33_ (C_I_)) or Se termination (Cu_2_Cd_3_1Se_33_ (C_II_), the spin-up and spin-down states are asymmetric, as shown in Figure 3d–e. Such electronic characters indicate that the systems contain some magnetism, which further indicates the oxidation state of Cu is Cu^2+^. It should be noted that the Cu impurity energy levels in both Cu_2_Cd_31_Se_33_ (C_I_) and Cu_2_Cd_31_Se_33_ (C_II_) reside about 0.43 eV and 0.46 eV above from the valence band maximum (which is very close to the Fermi level), respectively. These impurity levels correspond to the lower half of the forbidden gap (i.e., E_g/2_ = 0.93 eV and 1.12 eV, where Eg is bandgap) indicative of p-type dopants, which is evidence for the +2 oxidation state of Cu [18]. In addition, the behavior of Cu^2+^ orbitals in our calculations is similar to the experimental observation of Cu^2+^ level in Cu-doped ZnSe NCs in which it was in the bandgap about 0.3–0.4 eV above the top of the valence band [50]. To further analyze the oxidation state of Cu in the QD, Bader charge calculations are performed for all Cu-doped Cd_33_Se_33_. A positive value of a Bader charge means the loss of electrons; otherwise, it means the gain of electrons. As shown in Table 3, the charge state of Cu ions in Cu_2_Cd_31_Se_33_ (C_I_) and Cu_2_Cd_31_Se_33_ (C_II_) is about 0.084–0.129 |e| more positive than that for Cu ions in CuCd_32_Se_33_ (S), CuCd_32_Se_33_ (C_I_), CuCd_32_Se_33_ (C_II_), and Cu_2_Cd_31_Se_33_ (C_III_), suggesting that Cu ions lose more electrons in Cu_2_Cd_31_Se_33_ (C_I_) and Cu_2_Cd_31_Se_33_ (C_II_) than in other cases. This is consistent with that the oxidation state of Cu^2+^ in Cu_2_Cd_31_Se_33_ (C_I_) and Cu_2_Cd_31_Se_33_ (C_II_) is larger than the oxidation state of Cu^1+^ in other CdSe QDs.

Combined with the calculated binding energy in Section 3.2, it seems that the system with Cu^+^ ions (CuCd_32_Se_33_ (S), CuCd_32_Se_33_ (C_I_), CuCd_32_Se_33_ (C_II_), Cu_2_Cd_31_Se_33_ (C_III_), and Cu_2_Cd_31_Se_33_ (S)) is more stable than that with Cu^2+^ impurity ions (Cu_2_Cd_31_Se_33_ (C_II_)) because the former has larger binding energies than the later (Table 2). Our results are consistent with previous studies in which the oxidation state of Cu (+1) is more stable than Cu (+2) in some ZnS and CdS hosts [32,51].

### 3.4. Electronic Properties for Cu-Doped Cd_33_Se_33_ Quantum Dots

**Figure 4:**
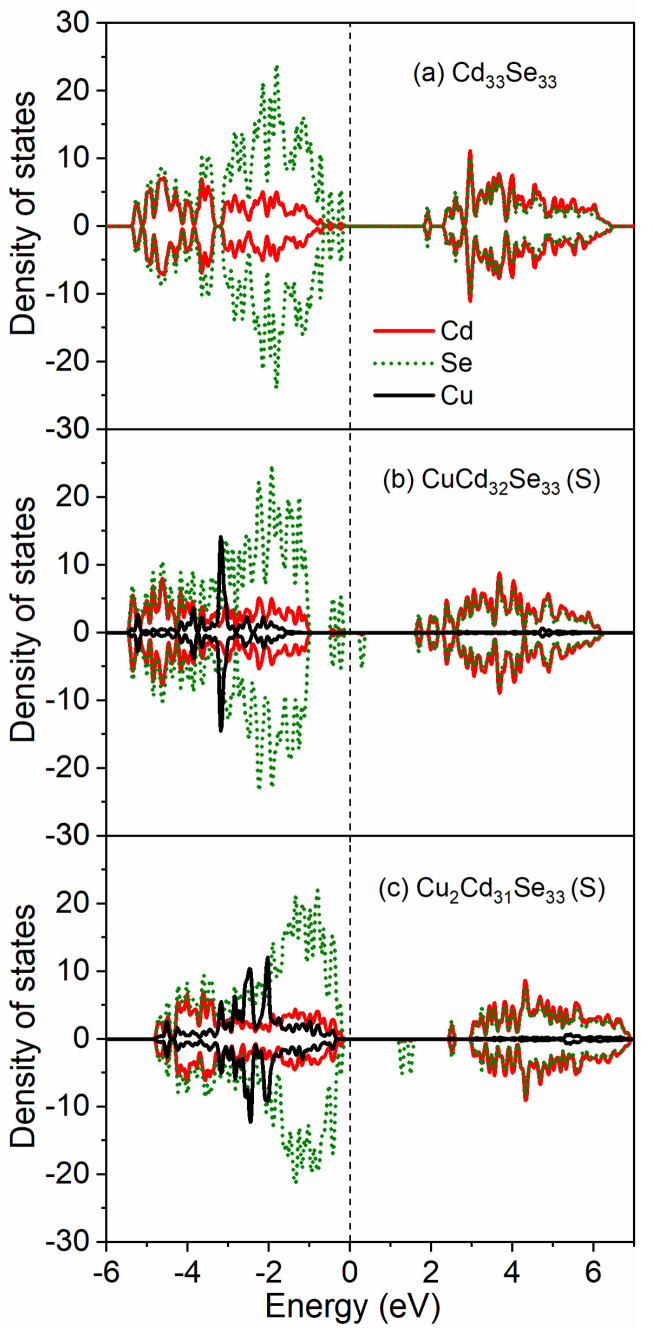
Projected density of states (DOS) for each type of ion when Cu atoms are doped at the surface of Cd_33_Se_33_: (**a**) pristine Cd_33_Se_33_; (**b**) CuCd_32_Se_33_ (S); (**c**) Cu_2_Cd_31_Se_33_(S). The Fermi level is located at 0 eV.

**Figure 5:**
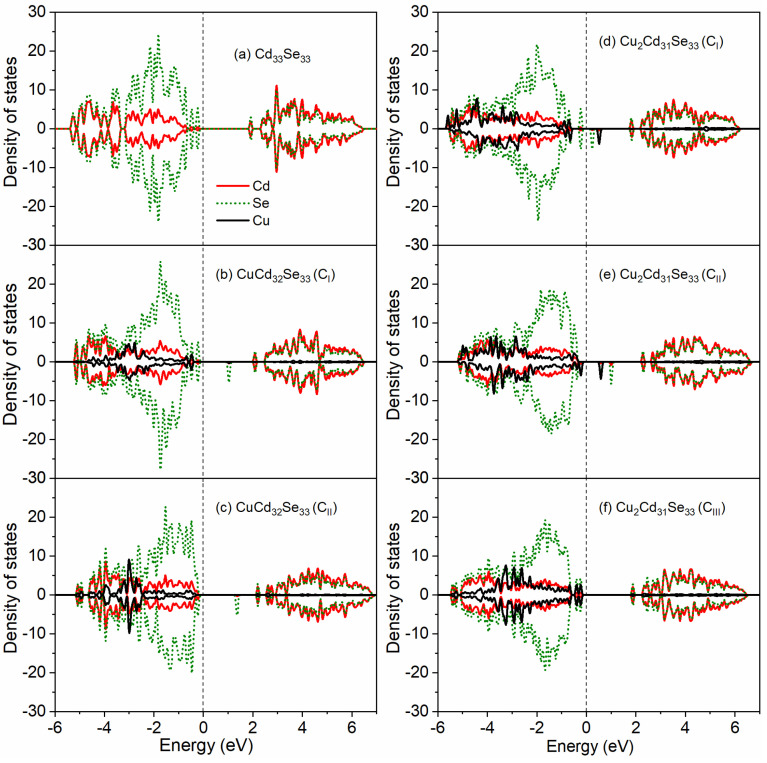
Projected density of states (DOS) for each type of ion when Cu atoms are doped in core region of Cd_33_Se_33_: (**a**) pristine Cd_33_Se_33_; (**b**) CuCd_32_Se_33_ (C_I_); (**c**) CuCd_32_Se_33_ (C_II_); (**d**) Cu_2_Cd_31_Se_33_(C_I_); (**e**) Cu_2_Cd_31_Se_33_(C_II_); (**f**) Cu_2_Cd_31_Se_33_(C_III_). The Fermi level is located at 0 eV.

To explore how Cu impurity influences the electronic structure of Cd_33_Se_33_, the density of states with and without Cu dopants are presented in Figure 4 for surface doping and Figure 5 for core doping, respectively. For the pristine Cd_33_Se_33_ (Figure 4a), previously we have demonstrated [48] that the mid-gap states primarily consist of Se 4p orbitals locating above the valence band (VB); the conduction band is mainly composed of Cd 5s and Se 4p states. With one Cu dopant incorporated into the surface (Figure 4b), the mid-gap states, which mainly consist of Se 4p states from two-coordinated surface Se atoms, are located on the top of the valence band. The Cu 3d orbitals in the VB are located about 1.35 eV below the Fermi level so that they do not contribute to the mid-gap states. In the forbidden band, a new Se 4p impurity state above Fermi level emerges. With the increasing Cu dopants on the surface (Figure 4c), the mid-gap states above the VB disappear and Cu 3d orbitals shift toward the top of the valence band. The impurity levels still exist in the forbidden gap but shift toward the conduction band minimum.

The incorporation of Cu dopants into the core region (Figure 5) results in very different electronic structures from those on the surface, but these doped NCs still maintain the insulating character. Such an insulating character for core-doped Cu:Cd_33_Se_33_ is in drastic contrast to the metallic-like character for core-doped Ag:Cd_33_Se_33_ in which some electrons are located at the Fermi level of the system [48]. In CuCd_32_Se_33_ (C_I_) shown in Figure 5b, the mid-gap states consisting of mainly Se 4p orbitals and slightly Cu 3d orbitals are just located above the valence band. Hybridization of Cu 3d orbitals with Se 4p states appears above the VB and both of them contribute to the mid-gap states. Similar hybridization electronic states above the VB have been also observed when a Cu impurity replaces a central Cd atom in zinc-blende-based CdSe NCs (note our NCs are based on the Wurtzite structure), although they consist of Cu 3d orbitals primarily [52]. The energy levels of dopant d orbitals are deeper than the anion p states in VB showing the “inverted” bonding character in CuCd_32_Se_33_ (C_I_). This is different from the “normal” bonding feature (in which the dopant d orbitals are located at shallower energy levels than the anion p states in VB) for a Cu impurity locating on the center of a zinc blende (ZB) based CdSe nanocrystal [52]. When two Cu dopants are incorporated into the core region near the Cd-terminated (Cu_2_Cd_31_Se_33_ (C_I_)) shown in Figure 5d, the mid-gap states are still above the VB but without the contribution from Cu 3d orbitals. However, as the Cu dopants are close to the Se-terminated (CuCd_32_Se_33_ (C_II_) and Cu_2_Cd_31_Se_33_ (C_II_)), the mid-gap states disappear, as can be seen in Figure 5c,e. More interestingly, when Cu dopants are near both Se-terminated and Cd-terminated in Cu_2_Cd_31_Se_33_ (C_III_), the mid-gap states with the hybridization of Cu 3d and Se 4p orbitals appear above the VB (Figure 5f). As for the conduction band of core-doped Cu:Cd_33_Se_33_ QDs, an impurity level consisting of Se 4p states locates within the forbidden band in CuCd_32_Se_33_ (C_I_) (Figure 5b) and CuCd_32_Se_33_ (C_II_) (Figure 5c). With the increasing Cu dopants, an additional impurity level composed of Cu 3d orbitals appears within the forbidden band for Cu_2_Cd_31_Se_33_ (C_I_) (Figure 5d) and Cu_2_Cd_31_Se_33_ (C_II_) (Figure 5e). This is consistent with the electronic character of Cu^2+^ as described in Section 3.4. However, in the case of Cu_2_Cd_31_Se_33_ (C_III_) (Figure 5f), there are no impurity levels. The above electronic structure analysis demonstrates that the energy levels and shapes of the valence and conduction bands can be modified by changing Cu concentrations and locations in CdSe QDs, which will, in turn, affect the optical absorption properties of these QDs as discussed in the next section.

### 3.5. Optical Absorption Spectra for Cu-Doped Cd_33_Se_33_ Quantum Dots

**Figure 6:**
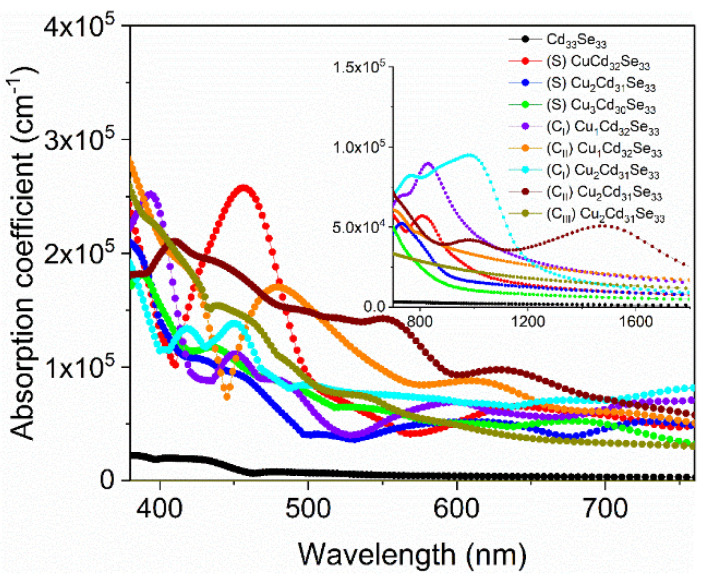
Absorption coefficients as a function of wavelength for Cd_33_Se_33_ QDs without and with Cu doping in the visible region in the main figure. The inset shows the absorption coefficients of these QDs in the infrared region.

To explore how the copper incorporation affects the optical properties of Cd_33_Se_33_ QDs, the optical absorption spectra of Cd_33_Se_33_ with and without Cu dopants are calculated within the independent-particle approximation, which is known to provide a qualitative agreement of dielectric response functions between theory and experiment while neglecting self-energy, excitonic and local-field effects in the optical response [53,54]. For the Cd_33_Se_33_ QD, the calculated lowest absorption peak is in good agreement with the experiments [30,55]. Clearly, doping of Cu in the Cd_33_Se_33_ leads to different absorption spectra from the pristine one, as can been seen in Figure 6. In the visible region (the main figure of Figure 6), just one Cu dopant induces high-intensity absorption peaks in the range of 408–502 nm for CuCd_32_Se_33_ (S), 380–428 nm for CuCd_32_Se_33_ (C_I_), and 445–574 nm for CuCd_32_Se_33_ (C_II_). In experiments, the enhancement of absorption spectra in the wavelength range between 390 and 600 nm was also observed in Cu-doped CdSe NCs [19,22,31]. When two or three Cu atoms are doped, absorption peaks are relatively weaker within the same wavelength range. These results indicate that the optical intensity of Cu-doped Cd_33_Se_33_ in the visible region strongly correlates with Cu dopant content. A similar dopant–concentration-dependent trend in optical absorption was also observed in Ag-doped CdSe QDs [48]. These new absorption features result predominantly from the electronic transition from Se 4p orbitals in the valence band to Se 4p and Cd 5s states in the conduction band (as shown in Figure 4 and Figure 5).

In the infrared regime (displayed in the inset of Figure 6), the Cu_2_Cd_31_Se_33_ (C_I_) exhibits a stronger absorption peak than other CdSe QDs between 815 and 1161 nm; The Cu_2_Cd_31_Se_33_ (C_II_) has a unique absorption peak from 1148 to 1777 nm. These absorption features are induced mainly by electronic excitations from Se 4p orbitals on the top of VB to Cu 3d orbitals in the forbidden gap (as indicated in Figure 5). As analyzed in Section 3.4, Cu dopants show the +2 oxidation state in Cu_2_Cd_31_Se_33_ (C_I_) and Cu_2_Cd_31_Se_33_ (C_II_) while exhibiting the +1 oxidation state in other QDs. The result indicates that incorporation of Cu^2+^ in Cd_33_Se_33_ QDs can cause stronger optical absorption in the infrared regime than Cu^+^. This can be explained by the fact that the 3d states of Cu^2+^ reside above the Fermi level and are near the valence band edge (Figure 5d,e). Such an electronic structure can lead to a small sub-bandgap electronic transition from VB to impurity levels and thus enhance optical absorption in the long-wavelength range.

Our results suggest that the incorporation of Cu can dramatically enhance the optical absorption of Cd_33_Se_33_ QD within the visible regime and extend the absorption edges into the infrared regime. Furthermore, the optical absorption of Cu-doped Cd_33_Se_33_ in the visible regime is affected mainly by Cu dopant concentration, while the absorption in the infrared regime is closely related to the oxidation state of Cu.

## 4. Conclusions

In this work, a systematic density functional theory (DFT) modeling using the Heyd–Scuseria–Ernzerhof (HSE) screened Coulomb hybrid functional was conducted to explore how the location and concentration of Cu dopants influence the structural, energetic, electronic, and optical properties of a magic-sized Cd_33_Se_33_ QD. It was found that the oxidation state of Cu dopants is closely related to their substitution location and concentration. Doping of Cu at the surface of Cd_33_Se_33_ QD leads to the Cu^+^ 3d orbitals distributions. Similar distributions of Cu^+^ 3d states were also observed for Cu in the core region such as CuCd_32_Se_33_ (C_I_) (Cu atom near the Cd-terminated), CuCd_32_Se_33_ (C_II_) (Cu atom near the Se-terminated), and Cu_2_Cd_31_Se_33_ (C_III_) (one Cu atom near the Cd-terminated and the other near the Se-terminated). However, doping of both copper atoms at internal Cd-sites near Cd-terminated or Se-terminated leads to the Cu^2+^ electronic character. Further binding energy calculations showed that in general, the Cu dopants with a +1 oxidation state are energetically more stable than those with a +2 oxidation state in Cd_33_Se_33_. It was also found that the optical absorption coefficient of Cu-doped Cd_33_Se_33_ in the infrared regime is closely related to the oxidation state of Cu rather than Cu concentration. On the other hand, doping of only one Cu atom introduces stronger absorption peaks than the doping of two Cu atoms in the range of 380–574 nm, indicating that the absorption coefficient is sensitive to Cu concentration in the visible regime. This work demonstrates that the dopant location and concentration can have significant effects on the electronic structure of Cu 3d states. Therefore, the proper control of the dopant concentration and position is critical for improving the efficiency and performance of CdSe QD-based optoelectronic devices.

## Figures and Tables

**Table 1:** The Cu-Se bond length (d<Cu-Se>) and Se-Cu-Se bond angle (∠Se-Cu-Se) between a Cu atom and its adjacent Se atoms in Cu-doped Cd_33_Se_33_ QDs. The Cd-Se bond length (d<Cd-Se>) in the core region (denoted as “C”), and Se-Cd-Se bond angle (∠Se-Cd-Se) in the surface region (denoted as “S”) for pristine Cd_33_Se_33_ are also presented as references, where Cd atom is the one replaced by Cu. The C_I_, C_II,_ and C_III_ are defined in Methodologies.

	d<Cd-Se> (Å)	d<Cu-Se> (Å)	∠Se-Cd-Se (°)	∠Se-Cu-Se (°)
Cd_33_Se_33_ (C)	2.678		109.04	
Cd_33_Se_33_ (S)	2.657		119.78	
CuCd_32_Se_33_ (S)		2.262		177.83
Cu_2_Cd_31_Se_33_ (S)		2.269		171.93
Cu_3_Cd_30_Se_33_ (S)		2.272		168.50
CuCd_32_Se_33_ (C_I_)		2.414		120.0
CuCd_32_Se_33_ (C_II_)		2.385		119.8
Cu_2_Cd_31_Se_33_ (C_I_)		2.442		113.28
Cu_2_Cd_31_Se_33_S (C_I_)		2.477		109.39
Cu_2_Cd_31_Se_33_ (C_II_)		2.522		109.85
Cu_2_Cd_31_Se_33_S(C_II_)		2.486		104.85
Cu_2_Cd_31_Se_33_ (C_III_)		2.451		112.51

**Table 2:** The total binding energy of Cu atoms and the binding energy per Cu atom for Cu in Cd_33_Se_33_. The location where Cd atoms are substituted by Cu is indicated in the parenthesis. The S, C_I_, C_II,_ and C_III_ are defined in Methodologies.

	Total Binding Energy (eV)	Binding Energy/Cu Atom (eV)
CuCd_32_Se_33_ (S)	1.824	1.824
CuCd_32_Se_33_ (C_I_)	1.726	1.726
CuCd_32_Se_33_ (C_II_)	1.466	1.466
Cu_2_Cd_31_Se_33_ (S)	0.538	0.269
Cu_2_Cd_31_Se_33_ (C_I_)	0.267	0.134
Cu_2_Cd_31_Se_33_ (C_II_)	0.497	0.248
Cu_2_Cd_31_Se_33_ (C_III_)	0.797	0.398
Cu_3_Cd_30_Se_33_ (S)	−0.397	−0.466

**Table 3:** Average Bader charge (|e|) for each ion in Cu-doped Cd_33_Se_33_ QDs. The S,C_I_,C_II_ and C_III_ are defined in Methodologies.

	Cu	Cd	Se
CuCd_32_Se_33_ (S)	0.204	0.647	−0.634
CuCd_32_Se_33_ (C_I_)	0.288	0.671	−0.660
CuCd_32_Se_33_ (C_II_)	0.261	0.671	−0.659
Cu_2_Cd_31_Se_33_ (S)	0.266	0.673	−0.648
Cu_2_Cd_31_Se_33_ (C_I_)	0.345	0.674	−0.654
Cu_2_Cd_31_Se_33_ (C_II_)	0.390	0.670	−0.653
Cu_2_Cd_31_Se_33_ (C_III_)	0.287	0.670	−0.647
Cu_3_Cd_30_Se_33_ (S)	0.260	0.667	−0.630

## Data Availability

The data presented in this study are available on request from the corresponding author. The data are not publicly available due to ethical.
